# Photoperiod-Induced Neuroplasticity in the Circadian System

**DOI:** 10.1155/2018/5147585

**Published:** 2018-02-28

**Authors:** Alessandra Porcu, Malini Riddle, Davide Dulcis, David K. Welsh

**Affiliations:** ^1^Department of Psychiatry and Center for Circadian Biology, University of California, San Diego, 9500 Gilman Dr., San Diego, CA MC-0603, USA; ^2^Veterans Affairs San Diego Healthcare System, San Diego, CA, USA

## Abstract

Seasonal changes in light exposure have profound effects on behavioral and physiological functions in many species, including effects on mood and cognitive function in humans. The mammalian brain's master circadian clock, the suprachiasmatic nucleus (SCN), transmits information about external light conditions to other brain regions, including some implicated in mood and cognition. Although the detailed mechanisms are not yet known, the SCN undergoes highly plastic changes at the cellular and network levels under different light conditions. We therefore propose that the SCN may be an essential mediator of the effects of seasonal changes of day length on mental health. In this review, we explore various forms of neuroplasticity that occur in the SCN and other brain regions to facilitate seasonal adaptation, particularly altered phase distribution of cellular circadian oscillators in the SCN and changes in hypothalamic neurotransmitter expression.

## 1. Introduction

To adapt to changes in the light-dark environment, organisms have evolved an internal circadian (ca. 24 hr) clock that drives many behavioral and physiological outputs, such as daily cycles of sleep-wake, metabolism, hormone secretion, and cognitive function. Light, the circadian clock, and output behaviors interact closely to produce a temporal order that is essential for survival [1]. In mammals, the principal circadian pacemaker is the bilateral suprachiasmatic nucleus (SCN), a deep brain nucleus located dorsal to the optic chiasm in the ventral periventricular zone of the hypothalamus [2, 3]. Innervated by glutamatergic retinal afferents emanating from the optic chiasm, the SCN is the first brain structure to receive photic information [2]. The SCN synchronizes itself to external light-dark cycles according to light-dependent signals transmitted via the retinohypothalamic tract [4] and then communicates timing information to peripheral clocks through various output pathways [5]. At the molecular level, circadian rhythms are controlled by an autoregulatory transcriptional-translational negative feedback loop within individual cells. At dawn, the transcription factor circadian locomotor output cycles kaput (CLOCK) and brain and muscle ARNT-like protein 1 (BMAL1) form complexes and bind DNA in a specific promoter region (E-Box) to activate the transcription of target genes, including the *Period* (*Per1*, *Per2*, and *Per3*) and *Cryptochrome* (*Cry1* and *Cry2*) gene families. In turn, PER and CRY proteins form complexes that translocate into the nucleus to repress CLOCK/BMAL1-mediated transcriptional activation [6]. An additional regulatory loop includes both positive and negative regulatory elements: retinoic acid-related orphan receptor ROR (*α*, *β*, and *γ*) and nuclear heme receptor REV-ERB (*α* and *β*), respectively [7, 8]. These molecular rhythms result in appropriately timed cycles of physiology, metabolism, and behavior.

For decades, the SCN has been described as a circadian pacemaker transmitting one integrated, rhythmic signal to the rest of the body. Many studies have shown, however, that the SCN is composed of many cells with different properties and that each cell has unique inputs and downstream targets. SCN neurons coordinate with one another to adapt the nucleus to different light environments, leading to highly plastic changes at the cellular and network levels. These changes may influence the activity of SCN targets.

The highly plastic nature of SCN cells and networks may explain why many physiological and behavioral processes are affected by short or long photoperiods and why many neurological disorders are associated with irregular light environments. In this review, we will discuss recent studies that have investigated the influence of photoperiod on the SCN and other brain regions. We will highlight studies that broaden our understanding of neuronal phenotype plasticity and related effects on circadian rhythms and behavior.

## 2. Seasonal Adaptation

In addition to its role as a circadian pacemaker, the SCN also mediates photoperiodism, a process whereby organisms gradually adapt to the length of the daily light period (photoperiod) and track time-of-year with seasonal changes in physiology and behavior [9]. The SCN also relays photic signals to brain regions implicated in sleep, mood, and motivational states [10], allowing for more acute regulation of these processes by light. It is clear that changes in light input have profound effects on the functionality of many brain systems, especially those mediating reproductive function in seasonally breeding species such as sheep or hamsters [11]. In humans, alterations in the light environment, such as short days during winter, are associated with mental health deficits, including seasonal affective disorder (SAD) [12] and cognitive dysfunction [13]. Moreover, changes in light-dark cycles can induce anxiety- and depression-like behaviors in both diurnal (day-active) and nocturnal (night-active) adult rodents [12, 14, 15]. These findings suggest the possibility of an SCN-mediated effect of photoperiod on mental health.

Individual SCN neurons demonstrate circadian rhythms of electrical activity with a wide range of intrinsic periods (from 22 h to 28 h) [16–18]. SCN cells are autonomous single-cell oscillators driven by an intrinsic molecular feedback loop [16]. However, to encode photoperiodic information, the presence of a functional neuronal network among SCN cells is necessary [19]. The response of the SCN to light and its ability to regulate seasonal rhythms are dependent upon synaptic plasticity among neurons of the SCN's network [20]. By adjusting the phase relationship among single cell oscillators, the SCN can code for short days or long days. This circadian plasticity can then modulate physiological functions to respond to environmental photoperiodic changes. Fine tuning of multioscillatory molecular machinery in clock neurons and daily changes at the cellular or network level are the main features of circadian plasticity.

Melatonin, the “darkness hormone” produced by the pineal gland, is not only a prominent rhythmic output of the SCN mediating downstream photoperiodic responses but also plays a key feedback role in modulating function of the SCN, which contains a high density of melatonin receptors [21]. In providing rhythmic feedback to the SCN circadian clock, melatonin has both immediate and long-term effects. Upon receptor binding, melatonin generates an outward potassium current suppressing SCN neuronal activity during night time [22]. Moreover, melatonin decreases VIP-induced and spontaneous release of AVP in the SCN in nocturnal rats [23]. In the long term, melatonin induces phase-dependent phase shifts and amplifies circadian rhythmicity in the SCN. Despite numerous studies reporting effects of melatonin on circadian rhythms in rodents, its exact role in SCN feedback has not yet been clarified. However, it is clear that under natural conditions in photoperiodic species, there are seasonal variations in duration of nocturnal melatonin secretion, with longer durations in winter [24], and that these seasonal variations are SCN-dependent. Light can affect melatonin rhythms; in particular, increased exposure to evening light can delay phase of melatonin onset in spring and summer [25]. The observation that melatonin rhythms are altered in patients with SAD [26] demands further investigation of the role of melatonin in modulating circadian plasticity and its effect on mood.

Neurons in many parts of the brain (not only the SCN) show functional adaptation in response to a change in photoperiod. One form of neuronal plasticity is neurotransmitter respecification, which alters synaptic function and can influence behavior [27, 28]. For decades, it had been assumed that the type of neurotransmitter expressed by a specific class of neurons remains stable and immutable throughout life once it is specified early in development. However, recent evidence indicates that transmitters expressed by neurons can be changed both in the developing [29] and in the adult brain via alterations in gene expression, intercellular interactions, and calcium-mediated electrical activity [30]. Interestingly, Dulcis et al. [14] have found that altered photoperiod can induce neurotransmitter respecification via somatostatin-to-dopamine switching in the rat hypothalamus, with concomitant changes in stress-response behavior. Importantly, summer and winter photoperiods have also been associated with changes in the total number of dopaminergic neurons in humans [31]. The presence of light-induced neurotransmitter switching in the adult rat PVN [32] suggests the possibility of a similar process in the SCN. The high rate of neurotransmitter coexpression displayed by SCN neurons even at baseline (typically GABA plus one or more neuropeptides) [33] suggests that they may already have in place whatever molecular machinery is required to express multiple neurotransmitter phenotypes [28].

## 3. SCN Light Input Circuits

**Figure 1:**
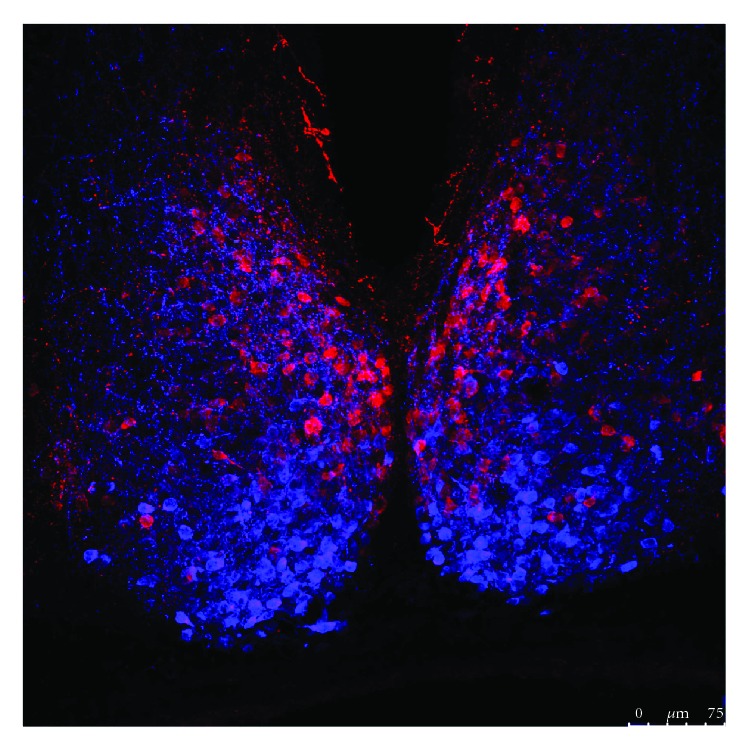
Representative confocal microscopic image of neuropeptide expression in the mouse suprachiasmatic nucleus (SCN). Slice was obtained from a CD1 mouse and processed for double immunofluorescence labeling for AVP (red) and VIP (blue). Scale bar 75 *μ*m. AVP: arginine vasopressin; VIP: vasoactive intestinal peptide.

The SCN is a bilaterally paired nucleus, located just above the optic chiasm in the anteroventral hypothalamus, that can be anatomically divided into ventral (core) and dorsal (shell) regions (Figure 1) [34, 35]. The SCN is comprised of ~20,000 tightly compacted, small-diameter neurons. The retinorecipient SCN core is made up of light-responsive neurons that receive glutamatergic input via the retinohypothalamic tract (RHT) [36, 37]. The initial step in glutamate transmission is activation of NMDA receptors, which trigger an increase in intracellular Ca^2+^ levels and stimulation of downstream signaling pathways. The transient activation and rapid phosphorylation of the Ca^2+^-cAMP response element-binding protein (CREB) initiate transcription of the *Per1* gene, as well as other CRE-regulated genes [38–40]. Interestingly, in other hypothalamic neurons, glutamate and voltage-gated calcium channels can play a key role in neurotransmitter switching [27]. Neurons within the SCN core that receive retinal input may express the neuropeptide vasoactive intestinal peptide (VIP) and/or gastrin-releasing peptide (GRP), as well as the small molecule neurotransmitter gamma-aminobutyric acid (GABA) [41]. Viewing the SCN sagittally confirms these patterns: VIP and GRP cells labeled in sagittal sections have been shown to receive dense RHT innervation, whereas arginine vasopressin (AVP) cells lying close to the core receive input from relatively few RHT fibers [42]. Core neurons are thought to synchronize the SCN shell to the external light-dark cycle through abundant local neuronal connections [43].

Neurons of the dorsal SCN shell generate robust circadian oscillations of gene expression [44, 45]. Shell neurons express neuropeptides such as AVP or prokineticin 2 (PK2), as well as GABA. While many studies have shown that RHT fibers are located primarily in the ventral core region, Morin and Allen [46] found more modest RHT innervation throughout the entire SCN. Corroborating studies using transgenic mice to label intrinsically photosensitive retinal ganglion cells (ipRGCs) confirmed projections to both core and shell subregions [47, 48]. Using triple-label immunohistochemistry in SCN sagittal sections, a recent study showed a regionally distinct pattern of RHT innervation [42]. Shell neurons are thought to play a major role in sending synchronizing output signals to peripheral clocks [49].

Core SCN neurons also receive projections from the retinorecipient intergeniculate leaflet of the thalamus and from the raphe nuclei [46]. These projections contribute importantly to the synchronizing effects of nonphotic stimuli on the SCN. It would be of interest to assess the potential role of these afferents in SCN circadian plasticity in response to nonphotic cues.

SCN cells have been shown to project from both core and shell subpopulations to other hypothalamic regions, including the subparaventricular zone [50]. These hypothalamic relay nuclei subsequently send projections widely throughout the brain to affect the nervous and endocrine systems. Considering the broad reach of the SCN and the anatomical complexity of its multisynaptic output, it is challenging to delineate the pathways by which the SCN network conveys light information to the rest of the brain and body.

In addition to their projections to the SCN, ipRGCs make connections to other brain regions such as the ventrolateral preoptic area (VLPO) and lateral hypothalamus, which are important for sleep regulation, and lateral habenula and amygdala, which are known to control mood [48]. These areas also receive photic signals indirectly through SCN projections. Light information converging from ipRGCs and the SCN at specific times of day may affect the physiological functions of these areas, in particular modulating mood and sleep [51]. This presents the possibility that the SCN, in addition to functioning as a master clock, can influence behavior by regulating light input to these brain regions.

Astrocytes are important cellular components within the SCN, implicated in circadian clock function, regulation of synaptic transmission, and intercellular coupling [52, 53]. Astrocytes play a critical role in the clearance of extracellular glutamate via glutamate transporters and activity of glutamine synthetase (GS) that converts glutamate to glutamine [54, 55]. Both the glutamate transporter (GluT) and glutamate enzymes are expressed in the SCN. An elegant study performed by Leone et al. [56] found that astroglial glutamate uptake in the mouse SCN is enhanced by daytime light, and glutamate is degraded by prolonged daytime GS activity. GS activity exhibits a circadian rhythm, and the presence of light and/or modulatory input from the eyes regulates glutamate metabolism in the SCN.

Differences between core and shell responsiveness to photic input can influence how the SCN network changes under different light conditions. A change in photoperiod has been shown to have a reorganizing effect on SCN networks: exposure to a long-day photoperiod leads to greater phase advance in rhythms of clock gene expression in core cells as compared to shell cells, resulting in desynchrony between the core and shell regions. This desynchrony leads to decreases in both shell rhythmic output and clock gene rhythm amplitude in downstream tissues [49]. Overall, it appears that a change in photoperiod temporarily diminishes the ability of the SCN to function as a pacemaker until core and shell regions are synchronous once again.

The ability of the SCN to adjust to a change in photoperiod by altering the relative circadian phase relationship among its component cellular oscillators is an unconventional form of neuronal network plasticity, revealed in SCN neuron circadian firing patterns as well as patterns of rhythmic clock gene expression. Electrophysiological recordings of multiunit activity in the SCN of freely moving mice reveal that the temporal distribution of neuronal firing activity is dependent upon photoperiod: in long days, the circadian peak of neuronal firing is broad, while in short days it is more compressed. Exploring the single-unit activity of SCN cells in vitro, VanderLeest et al. [32] found that firing peaks of single cells generally remain narrow and concluded that the broader population peak observed in long days arises from a wider phase distribution of the firing peaks of individual cells. Both in vitro and in vivo studies of electrical activity in the SCN reveal a decompression of the circadian peak in long days and a compression in short days, indicating that the temporal distribution of single-cell activity patterns defines the waveform of SCN rhythms (reviewed in Coomans et al. [19]). These changes may be more pronounced in the dorsal SCN than in the ventral SCN [57], and the detailed mechanisms by which they occur are unknown. However, it is now clear that plasticity in circadian phase distribution among SCN neurons is the primary mechanism encoding seasonal adaptation, notably via photoperiod-dependent changes in duration of nocturnal secretion of the pineal hormone melatonin [32].

Together, these findings suggest that environmental light conditions may influence mood and behavior through the SCN as well as other brain regions that receive ipRGC afferents. In the SCN, seasonal changes in photoperiod induce plastic changes in the relative phasing of cellular circadian oscillators, which in turn dictates the pattern of nocturnal melatonin secretion but is also modulated by melatonin feedback. The seasonally varying pattern of integrated rhythmic output from the SCN then influences other brain regions that are more directly involved in mood and cognitive function. This complex form of SCN neuronal plasticity thereby contributes to photoperiodic changes in many behavioral and physiological functions.

## 4. SCN Neurotransmitters Involved in Photoperiod-Induced Plasticity

VIP, GABA, and other peptide transmitters are known to be involved in maintaining synchronization among SCN neurons and producing a coherent circadian rhythm. The same transmitters also play a role in the phase plasticity that underlies seasonal encoding.

VIP neurons in particular play an important role in SCN neuron synchronization and photic signaling, and VIP expression is influenced by light [58, 59]. VIP-expressing cells in the ventral SCN receive light information from the retina and communicate through their projections to the dorsal SCN [41, 60]. Siberian hamsters housed under short-day conditions show an overall decrease in VIP expression throughout the circadian cycle relative to those housed under long-day conditions [61]. In vivo and in vitro application of VIP can mimic light-induced responses of the SCN [62, 63]. Genetic ablation of VIP or its receptor produces clear deficits in SCN cell synchronization in vitro [64]. Moreover, Lucassen et al. [65] have revealed critical roles for VIP in the SCN by in vivo electrophysiological measurements in freely moving VIP knockout mice. When transferred from a 12 : 12 hour light/dark cycle to constant darkness, VIP knockouts demonstrated a greater decrease of amplitude in multiunit neural activity (MUA) and behavioral rhythms relative to wild-type mice. Additionally, VIP knockouts did not adapt to a change in photoperiod. Together, these data imply a role for VIP in mediating intercellular coupling and encoding seasonal information. Further supporting these conclusions, Evans et al. [66] found that VIP signaling contributes to network synchronization in both steady-state and reorganized states during long-day exposure, acting as a cue transmitted from the SCN core to the SCN shell. Finally, a link between light input and synaptic remodeling has been reported using electron microscopy: the density of both glutamatergic and nonglutamatergic synapses on neurons expressing VIP is increased during the day, suggesting that the SCN can adapt continuously to environmental stimuli through rapid and reversible synaptic remodeling [67].

GABA and its biosynthetic enzyme, glutamic acid decarboxylase (GAD), are widely expressed in the SCN and affect its response to light. GABA_B_ receptors are thought to regulate photic signals by presynaptically modulating glutamatergic input from the RHT, whereas GABA_A_ receptors regulate local SCN circuits downstream of glutamatergic input [68, 69]. The influence of photoperiod on synaptic response to GABA has been evaluated using Ca^2+^ imaging and patch clamp electrophysiology in the SCN of mice exposed to long-day and short-day photoperiods. In mice exposed to long days, the polarity of GABA-evoked synaptic activity changes from inhibitory to excitatory in most SCN neurons [70]. Evans et al. [66] have shown that the frequency of spontaneous postsynaptic GABA-evoked currents decreases in short days, and photoperiod affects the equilibrium potential of GABA-evoked current, thus affecting GABA function. Relative to the long-day photoperiod, short days resulted in more inhibitory responses and fewer excitatory responses. These effects were restricted to the day, and no differences were observed in GABA-evoked responses at night. The increase of excitatory GABAergic activity observed in long days was reduced after blockade of the Cl^−^ cotransporter, suggesting a role of the cotransporter in seasonal adaptation.

These data suggest that photoperiod affects GABAergic activity by regulating cellular properties, perhaps thereby contributing to photoperiod-induced changes in phase distribution within the SCN network. Previous studies have suggested that the contribution of GABA in maintaining SCN synchrony is relatively modest [71]. However, Evans et al. [66] reveal that GABA_A_ signaling contributes to SCN coupling specifically when SCN core cells are already out of phase, demonstrating that GABA can either inhibit or promote network synchrony in a manner that depends on the dynamic state of the SCN network. In addition, GABA release in the SCN finely regulates diurnal rhythmicity of GABA_A_ receptor-expressing neurons by acting as an inhibitory transmitter at night and as an excitatory transmitter during the day [72]. Collectively, these studies indicate that SCN neuronal activity and photic signaling are regulated by GABA, which is influenced by daily light exposure. It is likely that the change in the polarity of the GABA response in the SCN is a form of plasticity that importantly shapes seasonal encoding [73].

GRP-expressing neurons are located in the ventral SCN, and there is clear evidence of their role in transmitting light information to other regions within the nucleus [74, 75]. Moreover, expression of GRP receptors within the SCN is influenced by light exposure [76], and in vitro application of GRP in SCN slices from VIP receptor-deficient mice promotes synchronization among SCN neurons. Further work testing whether GRP neurons may be involved in seasonal SCN network plasticity is warranted.

In contrast to VIP neurons, synaptic input to AVP-expressing neurons remains constant throughout the light-dark cycle [20]. However, daily rhythms of AVP expression are modulated by photoperiod. Rats exposed to long days show a longer interval of high AVP expression in the SCN compared to those exposed to short days [77]. SCN explants from hamsters housed under a long photoperiod display a higher peak level of AVP expression in vitro than do SCN from hamsters housed under short photoperiod [78]. Although it is known that AVP neurons project locally and exogenous AVP regulates the cellular activity of SCN neurons, only recently have studies revealed a role for AVP in SCN network function [79]. Mice with a *Bmal1* deletion specific to AVP neurons show diminished photoperiod response, with an increase in activity time under long days. Moreover, *Avp*-*Bmal1*^−/−^ mice show a reduction of *Per1* mRNA expression in the SCN after a 30 min light pulse, suggesting that deletion of *Bmal1* in AVP neurons may regulate the responsiveness of the ventral SCN to light [80]. Since AVP has been viewed as more of an SCN output signal [81, 82], it would be of interest to test the functional consequences of photoperiod exposure on the brain regions that receive primarily AVP afferents from the SCN.

Further investigation of how photoperiod may alter the expression of different neurotransmitters or neuropeptides in the SCN is needed for understanding mood disorders associated with seasonal changes in daily light exposure.

## 5. Clock Genes Reveal the Topography of Photoperiod-Induced Plasticity in the SCN

Rostral and caudal SCN neurons show differences in seasonal adaptation, as reflected in clock gene expression [83–86]. After exposing Siberian hamsters to either long or short photoperiod conditions, Hazlerigg et al. [85] found a difference between rostral and caudal expression of PER2, REV-ERB*α*, and D-site albumin promoter binding protein (Dbp) in the SCN. Under long days, all three genes showed a phase-advanced peak in the caudal SCN, while a slight phase delay was observed in the rostral SCN. No difference in peak expression was observed under short days. Similar asymmetry was observed in the electrical activity measured in SCN slices from Syrian hamsters [87]. Based on these findings, a model of two separate but mutually coupled oscillators was proposed in which caudal SCN neurons encode information regarding dawn, while rostral SCN neurons encode dusk [86].

**Figure 2:**
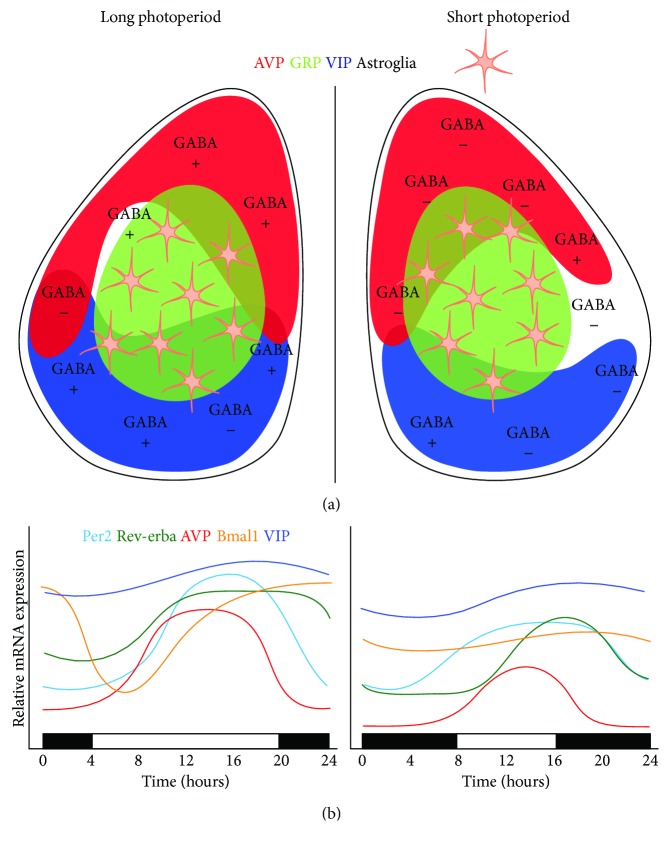
Proposed model for photoperiod-dependent cell rearrangement in the SCN. (a) Schematic of coronal SCN slice showing approximate spatial distributions of AVP, GRP, VIP, and astroglia. GABAergic inputs tend to result in excitatory responses under long photoperiod (left), while responses tend to be inhibitory under short photoperiod (right). (b) Relative mRNA expression levels of *Per2*, *Rev-erbα*, *AVP*, *Bmal1*, and *VIP* throughout a circadian cycle under long (left) photoperiod or short (right) photoperiod. Adapted from Ecker et al. [48] and Aton et al. [64]. AVP: arginine vasopressin; Bmal1: brain and muscle ARNT-like protein 1; GABA: gamma-aminobutyric acid; GRP: gastrin-releasing peptide; Per2: period 2; VIP: vasoactive intestinal peptide.

Using transgenic mice with a PER1 luciferase (PER1::LUC) reporter system, Inagaki et al. [84] found three different oscillating cell groups in the mouse SCN: one located in the caudal SCN and the other two in the rostral SCN. In vitro measurements of PER1 circadian rhythms in the SCN of mice previously exposed to long, intermediate, or short photoperiods showed that in the rostral SCN, the PER1 expression pattern is unimodal in short photoperiods, whereas a bimodal pattern is observed in long photoperiods. Surprisingly, PER2::LUC rhythms show a single cluster in both long and short photoperiod in the retinorecipient core SCN, suggesting differing roles for PER1 and PER2 in seasonal encoding [88]. Another study showed that exposure to long-day conditions induces a wider distribution of PER2::LUC peak times in the rostral SCN compared to short photoperiod, with larger period variability observed in the dorsolateral region [89]. Taken together, these data show that long-day exposure weakens intracellular coupling within SCN cells, leading to more heterogeneous phases of SCN neurons for seasonal encoding (Figure 2).

Exposure to a long photoperiod also alters expression of *Rev-erbα*, *Per2*, and *Clock* genes in the SCN of the European hamster. Interestingly, *Bmal1* mRNA expression appears clearly rhythmic under long days but is constitutively expressed under short days [78]. Further studies are needed to determine if the expression of the BMAL1 protein is also affected by photoperiod. However, *Per2*, *Rev-erbα*, and *Avp* genes, which are directly controlled by CLOCK:BMAL1 heterodimers [82, 90, 91], show decreased expression levels under short photoperiod, which may be a direct consequence of reduced transactivation by CLOCK:BMAL1 [78].

Clock genes play a role in epigenetic chromatin remodeling, which could contribute to seasonal plasticity in the SCN. CLOCK has histone acetyl transferase activity [92], and rhythmic CLOCK:BMAL1 DNA binding has been shown to promote rhythmic chromatin opening [93]. This indicates that the circadian feedback loop mediates the rhythmic regulation of chromatin accessibility and suggests that some CLOCK:BMAL1 effects could be mediated by the activity of other transcription factors. Photic stimulation can influence chromatin transitions between condensed and decondensed dynamic states in SCN cells [94], with resulting genetic alterations. For example, Azzi et al. [95] found that DNA methylation is necessary to temporally reorganize phase among SCN cells in mice exposed to different photoperiods. Furthermore, dorsal and ventral SCN cells exhibit methylation of different genes coding for neurotransmitter receptors and ion channels, including potassium, calcium, and GABA channels. Thus, photoperiod could act through epigenetic mechanisms to produce spatiotemporal reorganization of the SCN network. DNA methylation and histone acetylation may be a dynamic mechanism that contributes to neuronal plasticity for seasonal adaptation.

## 6. Photoperiod-Induced Plasticity in Other Brain Regions

**Figure 3:**
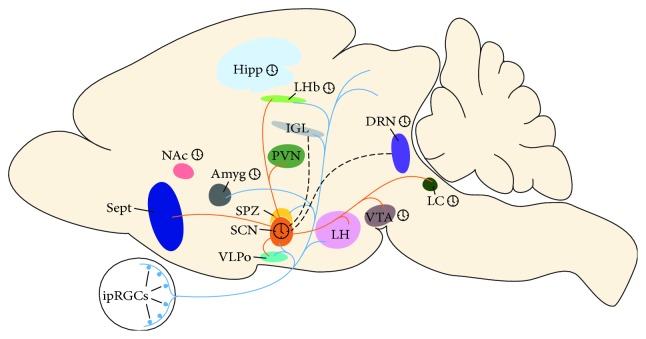
Schematic view highlighting certain ipRGC (blue) and SCN (orange) projections and areas that demonstrate rhythmic activity (clock symbol). Many areas receive input from both ipRGCs and the SCN, such as the VLPo, SPZ, LH, and LHb. The SCN receives input from the IGL and DRN (dashed lines). Amyg: amygdala; DRN: dorsal raphe nuclei; Hipp: hippocampus; IGL: intergeniculate leaflet; ipRGC: intrinsically photosensitive retinal ganglion cell; LC: locus coeruleus; LH: lateral hypothalamus; LHb: lateral habenula; NAc: nucleus accumbens; PVN: paraventricular nucleus; SCN: suprachiasmatic nucleus; Sept: septal area; SPZ: subparaventricular zone; VLPo: ventrolateral preoptic area; VTA: ventral tegmental area.

The SCN communicates light information to both hypothalamic and extrahypothalamic regions through efferent neuronal pathways (Figure 3). Several studies have identified six primary efferent projections from the SCN in the rat brain [96–99]. However, the existence of these pathways does not necessarily imply their use in communicating all types of photic information. For instance, in the Siberian hamster, cutting dorsomedial and dorsocaudal projections does not block melatonin signaling in short days, suggesting that these projections are not involved in transmitting photoperiod information from the SCN to other brain areas [100]. The SCN's paracrine function has been suggested as an additional output pathway; however, humoral signals diffusing from a transplanted SCN that can restore circadian rhythmicity are not able to restore the reproductive response to photoperiod [101, 102]. Together, these data suggest that different pathways are necessary to transmit circadian phase versus day length information from the SCN to other areas of the central nervous system.

Seasonal adaptation could also involve SCN-independent pathways through which light information is conveyed to brain regions that are directly innervated by ipRGCs. For example, an elegant study showed that an aberrant light cycle regulates mood and cognitive function in mice without substantially altering sleep or circadian timing. Light pulses at inappropriate times were shown to induce expression of the transcription factor c-Fos in the amygadala, lateral habenula, and subparaventricular nucleus, all of which are known to be ipRGC targets. Moreover, mice exposed to the aberrant light cycle demonstrated depression-like behavior and impaired hippocampal long-term potentiation and learning [103]. These data suggest that light can influence brain regions involved in mood and cognitive functions through direct ipRGC projections to these regions.

White-footed mice *(Peromyscus leucopus)*, small photoperiodic rodents, were found to have reduced hippocampal volume, impairments in hippocampal-mediated memory, and enhanced hypothalamic-pituitary-adrenal axis reactivity after short-day exposure [104, 105]. Moreover, a more recent study shows that short photoperiod enhances associative fear memory in auditory cue fear conditioning in these mice. These behavioral effects were associated with an increase in dendritic spine density of neurons in the basolateral amygdala [106]. This finding suggests that light could influence mood directly through ipRGC projections to the amygdala without altering the circadian system.

Dulcis et al. [14] found that the mammalian hypothalamus responds to different photoperiods by changing neurotransmitter identity. In particular, in the periventricular nucleus, the number of dopamine-releasing neurons increased in short days and decreased in long days, while the inverse was observed for somatostatin neurons. These two neuronal populations are mainly involved in the regulation of the stress response. Changing dopamine and somatostatin transmission resulted in altered anxiety and depression-like behaviors. In line with this work, another study found that rats exposed to long photoperiod exhibit depressive phenotypes in the forced swimming test and cognitive deficits in the novel object recognition task [107].

In contrast to nocturnal rats, crepuscular Siberian hamsters and diurnal sand rats [108] exposed to a short photoperiod show increased depressive-like behavior, as well as reduced soma size and dendritic complexity in the hippocampus CA1 region and increased spine density in the dentate gyrus. The observed dendritic changes were correlated with behavioral effects observed in the forced swim test, suggesting that alterations in the hippocampus in short days may be behaviorally relevant [109]. Depressed humans have reduced soma size in hippocampal CA1, CA2, and CA3 regions [110], which may account for the hippocampal volume reduction observed in humans with major depressive disorder.

Of interest, using a diurnal rodent model *(Arvichantis niloticus)*, Leach and collaborators found that decreasing daytime light intensity leads to depression-like behaviors, without affecting daily activity rhythms [111]. The depression-like behaviors were associated with a concomitant attenuation of serotonergic functions in dorsal raphe nucleus, periaqueductal gray, and medial cingulate cortex, brain regions known to be involved in mood regulation. These results demonstrate that decreased daytime light intensity, which mimics the transition from summer to winter in nature, induces depressive behaviors in diurnal grass rats. In summer, sunlight is brighter, and humans also spend more time outdoors (where the light is much brighter than indoors); consequently, light intensity may be as important as day length for regulating mood in humans.

Clock genes are expressed in cells throughout the brain, and some of these regions respond to light stimuli and generate SCN-independent circadian oscillations [112]. In mammals, responses to changing photoperiod have been observed especially in the hippocampus, one of the most plastic regions in the brain and also a key area that regulates emotion and cognition [113]. Moreover, seasonal affective disorder has been correlated with changes in hippocampal structure, and hippocampal gene expression shows daily rhythms regulated by the circadian system [114]. Siberian hamsters exposed to long days show diurnal rhythms in length of hippocampal dendrites, number of primary dendrites, and spine density, suggesting seasonal adaptation of hippocampal neuronal populations. Furthermore, *Bmal1* mRNA is highly expressed in the hippocampus, with increased circadian rhythm amplitude in short days compared to long days [115]. Thus, light input may be processed in the hippocampus through circadian expression patterns of clock genes.

In contrast to other studies in which light produces cognitive deficits in nocturnal rodents, Dellapolla et al. [116] found that long photoperiod enhances long-term recognition memory in mice, measured by spatial object recognition and novel object recognition tasks. Interestingly, whereas clock genes in the hippocampus were rhythmic in a neutral 12 hr photoperiod, rhythms of *Per1*, *Per2*, *Cry1*, and *Cry2* were reduced in long days. The authors suggest that long photoperiod suppresses the core clock mechanism in the mouse hippocampus, leading to de-repression of insulin-like growth factor II, which then enhances hippocampal function. These findings highlight a new way in which photoperiod may regulate cognitive function and suggest a key role of brain circadian clocks outside SCN in neuronal plasticity.

Finally, a number of variants in clock genes have been associated with mood disorders, including winter depression [117–119]. Recently, two rare missense variants in human *Per3* (PER3-P415A/H417R) were identified that cause familial advance sleep phase syndrome and are possibly associated with seasonal affective disorder. Transgenic mice carrying human PER3-P415A/H417R exhibit a lengthened circadian period in constant light and delayed phase in short-day conditions. At the molecular level, the mutation may exert effects on the clock by reducing PER3 stabilizing effects on PER1 and PER2. In the tail suspension test, PER3-P415A/H417R mice showed increased depression-like behavior, whereas *Per3*^−/−^ mice showed depression-like changes in various behavioral tests. These behavioral phenotypes were more evident in mice exposed to a short photoperiod, suggesting an effect of day length on depression-like behavior that may reflect seasonality traits in humans [117]. Xu et al. also found that 3 weeks of exposure to a shortened photoperiod combined with subchronic unpredictable stress significantly decreased sucrose preference and increased the levels of plasma melatonin, Neuropeptide Y, and corticosterone in adolescent male rats [120]. These data suggest that neuroendocrine changes may increase susceptibility to stress in adolescence induced by exposure to a short photoperiod. Together, these findings support the idea that clock genes may be important in mediating seasonal effects on mood.

## 7. Influence of Photoperiod during Early Development

Environmental stimuli, such as light, can affect neuronal development [29]. In the SCN, circadian rhythms in glucose utilization are present in the fetus, even though most connections among SCN cells develop postnatally [121]. Clock genes become rhythmic, with low amplitude, beginning at postnatal day 2 [122], and pups depend upon their mothers for photic synchronization until weaning. *Per1* and *Per2* expression are affected by altered light cycles at postnatal day 10, coinciding with the time when the RHT and connections to and from the SCN have developed adult features. It is only at P20–28, when the pups are weaned that the circadian system, becomes mature and independent from the mother's. Thus, the first 3 weeks after birth are critical for the development of the circadian system and its synchronization with the environmental photoperiod.

Previous studies have shown that seasonal photoperiods can imprint the circadian system. For example, using real-time gene expression and behavioral analysis, Ciarleglio and coworkers found that perinatal exposure to long days induced a narrower *Per1* expression waveform and shorter rhythm period compared to short-day exposure. This effect of perinatal photoperiod also applied to circadian behavior: mice that developed in long days also exhibited a shorter free-running period of locomotor activity rhythms [123]. Another group found that mice reared (P0–P21) under constant light (LL) showed a higher amplitude of the locomotor activity rhythm and lower levels of VIP and AVP in the SCN compared to mice reared under constant darkness (DD) or 12 L : 12 D light/dark cycles [124]. These results suggest that postnatal light experiences imprint the neuronal network that regulates seasonal adaptation by affecting the clock functions and output of SCN neurons. Recently, the same group found that mice raised in constant light and exposed to the same light condition as adults showed a shorter period and stronger circadian rhythms of body temperature compared to DD-raised mice and a tendency for depression-like behavior compared to 12 L : 12 D light/dark cycle-raised mice [125]. Moreover, compared to LD-raised mice, both LL- and DD-raised mice showed a decrease of glucocorticoid receptor expression in the hippocampus and increased plasma corticosterone concentration at the onset of the dark phase. Therefore, the postnatal light environment induces long-term effects on the hypothalamic-pituitary-adrenal axis and circadian system leading to a depressive phenotype in adulthood.

Interestingly, visual system development is influenced by light, and animals reared in DD show alterations in synaptic organization of the retina and in visual functions [126]. A recent study revealed that mouse eye development in utero is modulated by the fetal light response of melanopsin-expressing retinal ganglion cells [127]. Light induces retina tyrosine hydroxylase (TH) activity and dopamine production; retinal dopamine is the main factor coordinating retinal adaptation to changes in daily illumination [128]. In order to understand the potential effect of different photoperiods on retinal function, Jackson et al. exposed mice to summer- and winter-like light cycles during development and adulthood. Mice raised in short days showed enduring deficits in retinal light response and light-adaptive vision due to altered retinal dopaminergic signaling, including lower expression of TH, that are not reversed by increasing the amount of daily light in adulthood [129]. These results, together with the findings from the Dulcis group [129], show that photoperiod can regulate the activity and the phenotype of monoamine neurons in various brain regions, starting in the retina. Recent studies showed that patients with SAD exhibit lower retinal sensitivity and deficits in photic luminance response [130], which can be treated with light therapy, suggesting a mechanism of seasonal adaptation similar to that observed in mice.

As mentioned above, the intergeniculate leaflet receives serotonergic innervation from the dorsal raphe nucleus (DNR), and the SCN receives serotonergic input from the median raphe nucleus [131]. Moreover, DNR serotonin neurons innervate cortical and subcortical structures, which are known to regulate depression and anxiety states [132]. Maturation of these neurons in rodents can be modulated by environmental input in the first 3 or 4 weeks of postnatal life [133]. In particular, raising mice in long photoperiod increases the excitability and serotonin content of these neurons. The developmental effects of photoperiod on DNR serotonin neuronal firing rate were maintained even after reversal of photoperiod for 3–7 weeks in adulthood. Long photoperiod also increases midbrain monoamines and decreases anxiety- and depression-like behavior in a melatonin receptor 1-dependent manner [134]. Thus, photoperiod may regulate the maturation and physiology of serotonin neurons in the DRN via melatonin signaling, suggesting a possible mechanism for the observed variation in the risk of mood disorders in humans born at different times of the year [133, 135].

## 8. Conclusions

Our knowledge of the mechanisms by which the SCN and other brain regions adapt to seasonal changes in photoperiod is still incomplete. Traditional forms of neural plasticity include formation of new neural connections, changes in synaptic strength, and changes in synapse number. But in addition to these more familiar mechanisms, changing photoperiod can drive changes in phase relationship among cellular circadian oscillators in the SCN and in transmitter identity in other hypothalamic regions [136]. Further studies may reveal whether and how these two unconventional forms of neural plasticity might interact to mediate seasonal changes in behavior.
